# Medical Treatment for Ectopic Pregnancy during the COVID-19 Pandemic

**DOI:** 10.1055/s-0040-1718438

**Published:** 2020-12-21

**Authors:** Júlio Elito Júnior, Edward Araujo Júnior

**Affiliations:** 1 Department of Obstetrics, Escola Paulista de Medicina, Universidade Federal de São Paulo, São Paulo, SP, Brazil

Dear Editor,

The incidence of ectopic pregnancy is 2%, and during the COVID-19 pandemic, it became a challenge for obstetricians. 1 Ectopic pregnancy is the main cause of mortality in the first trimester of gestation. Therefore, early diagnosis and treatment should be performed to avoid morbidity and mortality. Early diagnosis is not common because pregnant women hesitate to seek medical assistance. Therefore, social isolation could postpone the diagnosis. To avoid this and encourage consultation, pregnant women should be informed of the risk factors for ectopic pregnancy, such as previous ectopic pregnancy, history of salpingitis, infertility, and others. On the other hand, patients with no risk factors should see a physician in case of first trimester bleeding.

Once diagnosis is confirmed, physicians should select the best applicable treatment, which should include outpatient management. As health systems are overburdened during the COVID-19 pandemic and personal protective equipment are limited, avoiding a surgical procedure becomes more necessary. 2

Methotrexate (MTX) treatment must be considered carefully, as incorrect dosage could lead to more risks for the patient. This may include failure of the treatment and a consequent emergent procedure for possible hemorrhage, as well as exposure of the patient to medical treatment with the potential risk of immunodeficiency. Therefore, medical treatment should be performed only in patients with the following inclusion criteria: unruptured ectopic pregnancy, hemodynamically stable, tubal pregnancy ≤3.5 cm, β-hCG levels ≤ 5,000 mUI/ml, and absence of embryonic cardiac activity detected by transvaginal ultrasonography. The exclusion criteria are as follows: intrauterine pregnancy; evidence of immunodeficiency, anemia, leukopenia, or thrombocytopenia; sensitivity to methotrexate (MTX); active pulmonary disease; active peptic ulcer disease; hepatic and renal dysfunction; decreasing levels of β-hCG at 24/48h before treatment; breastfeeding; and refusal to accept blood transfusion. 3

Once the patient was selected, the next step was to define the MTX regimen: single dose of MTX 50 mg/m ^2^ , two doses (50 mg/m ^2^ on days 1 and 4), multiple doses (MTX 1 mg/kg on days 1, 3, 5, and 7 and folinic acid 0.1 mg/mg on days 2, 4, 6, and 8). The single dose is the most commonly used regimen in patients with tubal pregnancy. Follow-up could be performed by telemedicine, with a focus on the β-hCG levels on days 4 and 7 after MTX regimen. If the levels decrease by more than 15% between days 4 and 7, the response is considered favorable. The patient should be followed up with β-hCG titers every week until the levels become negative. On the other hand, if the β-hCG level decrease by less than 15% between days 4 and 7, this indicates that an increase in MTX dose is necessary. 3

Expectant management can be performed in patients with declining β-hCG levels at an interval of 24/48 hours before treatment. The main criteria for expectant management are unruptured ectopic pregnancy, hemodynamically stable, declining levels of β-hCG at an interval of 24/48 hours, β-hCG levels ≤ 2,000 mUI/ml, tubal pregnancy diameter ≤ 3.5 cm, and absence of embryonic cardiac activity detected by transvaginal ultrasonography. 4 The surveillance is performed to check β-hCG levels every week until the titers become negative.

Surgery for tubal pregnancy should be performed in patients with ruptured tubal pregnancy, high levels of β-hCG, adnexal mass > 3.5 cm, and presence of live embryo. Patients diagnosed with COVID-19 should undergo laparoscopy because the MTX regimen could reduce immunity, and active pulmonary disease is a contraindication for MTX.

In most cases of non-tubal ectopic pregnancy (cervical, cesarean scar, interstitial and ovarian pregnancy), the standard treatment is surgery, such as hysterectomy. Some procedures alternative to surgery include local injection of MTX guided by transvaginal ultrasound, systemic medical treatment with MTX, and embolization of uterine arteries. Management in cases of interstitial, cervical, and cesarean scar pregnancies should always be on a case by case basis. When the embryo has a heartbeat, transvaginal ultrasonography-guided local treatment with MTX injection into the gestational sac at a dose of 1 mg/kg is the first-line treatment. Systemic treatment with MTX is performed in cases in which the embryo has no heartbeat. Medical treatment will depend on the initial β-hCG titer. For titers < 5,000 mIU/mL, a single dose of MTX 50 mg/m ^2^ is recommended. On the other hand, if β-hCG titers are > 5,000 mIU/ml, a multiple-dose MTX protocol is recommended.

In summary, clinical treatment of ectopic pregnancy by MTX or expectant management is an alternative during the COVID-19 pandemic. An early diagnosis and appropriate selection of treatment options are critical for the success of the treatment.
